# Diagnostic Value of Plasma Long Non-coding RNA HOTTIP as a Non-invasive Biomarker for Colorectal Cancer (A Case- Control Study)

**DOI:** 10.22088/IJMCM.BUMS.8.4.240

**Published:** 2019

**Authors:** Soheila Ali Akbar-Esfahani, Morteza Karimipoor, Fatemeh Bahreini, Ali Reza Soltania, Najme Aletaha, Ali Mahdavinezhad

**Affiliations:** 1 *Department of Molecular Medicine & Genetics, Hamadan University of Medical Sciences, Hamadan, Iran.*; 2 *Molecular Medicine Department, Biotechnology Research Center, Pasteur Institute of Iran, Tehran, Iran.*; 3 *Modelling of Non communicable Diseases Research Center, School of Public Health, Hamadan University of Medical Sciences, Hamadan, Iran.*; 4 *Department of Gastroenterology, Imam Khomeini Hospital, Tehran University of Medical Science, Tehran, Iran.*; 5 *Research center for Molecular Medicine, Department of Molecular Medicine and Genetics, Hamadan University of Medical Sciences, Hamadan, Iran. *

**Keywords:** Biomarker, colorectal neoplasms, long non-coding RNAs, lncRNA HOTTIP

## Abstract

Long non-coding RNAs (lncRNAs), associated with various cancers including colorectal cancer (CRC), could be collected from body fluids easily. Our aims were to determine the expression level of *HOTTIP* lncRNA in plasma samples of healthy individuals and CRC patients as well as their relationship with clinico-pathological characteristics of patients. First, total RNA was extracted from the plasma samples of 100 subjects including 50 patients and 50 age and sex matched healthy persons. Then, gene expression was measured using real-time PCR technique. The sensitivity and specificity of *HOTTIP* dysregulation in CRC and healthy individual’s plasma was measured by receiver operating characteristic (ROC) analysis. As compared with healthy controls, *HOTTIP* lncRNA was over expressed in a statistically significant manner in plasma samples of patients (P=0.001). Significant relationship between *HOTTIP* expression and positive family history of CRC was observed, too (P=0.04). The ROC curve analysis showed an AUC value of 0.775, a specificity of 82%, a sensitivity of 76%, with a cut off value equal to 2.40 (P =0.001). *HOTTIP* transcript can be proposed as a new biomarker for early diagnosis due to the increased expression in plasma samples of patients with CRC and the relatively high sensitivity and specificity.

Colorectal cancer (CRC) is ranked third among the most common malignant diseases world-wide (1). Contrary to the many advances in CRC treatment, patients’ survival rate is still changing to a limited extent due to the lack of an effective screening test (2). Age and stage at diagnosis are crucial factors that impress patients survival rate so that the lowest survival is attributed to stage IV of CRC affected patients (3). Therefore, what has attracted the attention of the scientists is finding noninvasive novel biomarkers to diagnose CRC in early stages and predict prognosis to choose an appropriate treatment which is an inevitable need.

Long noncoding RNAs (lncRNAs) are a class of transcribed RNA molecules with a length of greater than 200 nucleotides that are involved in various biological functions such as alternative splicing, chromatin remodeling, and mRNA degradation as well as cell cycle, survival and migration (4, 5). LncRNAs are smaller than mRNAs, contain less but longer exons, and are expressed relatively at lower levels. Most of these molecules represent a specific expression pattern in different tissues so that the aberrant expression of lncRNAs can cause various diseases, including CRC (1, 5-8).

The *HOTTIP *gene in the homeobox A (HOXA) locus is located at 7p15.2 chromosome and encodes a 4665-bp transcript (9). HOXA locus in mammals contains a cluster containing eleven *HOX* genes with a series of expression patterns ranging from proximal to distal. In general, HOXA transcript at the distal tip (*HOTTIP*) lncRNA is able to activate *HOX* genes by employing histone-modifying enzymes and silencing tumor suppressor genes (10-12). Recently, it was reported that *HOTTIP* is up regulated in CRC tissue samples compared with healthy adjacent tissues and it has a positive association with T and clinical stages (13). On the other hand, lncRNAs could be collected from body fluids easily (14). So the aims of our study were to determine the expression of *HOTTIP* in plasma samples of healthy individuals and CRC patients as well as their relationship with clinical and pathological characteristics of patients.

## Materials and methods

**Patient samples**

This case -control study was conducted on 100 plasma samples which fifty of them were obtained from patients suffering from CRC at stages I-III of disease regardless of age and sex with definite clinical and pathological status from Tumor Bank Department of Imam Khomeini Hospital, Tehran, Iran. The other 50 plasma samples were collected from suspected patients who underwent colonoscopy and their biopsy results were reported normal as control samples from the same hospital during 2017 which matched in age and sex with the patient group as much as possible. Following subjects were excluded from study groups: patients who received any treatment, cases with other organ cancers, history of chemotherapy and radiotherapy, systemic diseases such as renal failure, diabetes mellitus, and hypertension. Study protocol was approved by ethical committee of Hamadan University of Medical Sciences (Ethical code: IR.UMSHA.REC.1396.220). Participants signed written informed consent after explanation of the study purpose.

Patients included 22 women and 28 men with an average age of 60.98 ± 13.95 (29-85) and healthy people consisted of 24 women and 26 men with an average age of 59.26 ± 14.38 (22-85).The other clinicopathological information of the patients are shown in Table 1.

**Relative expression of** ***HOTTIP*** **transcript by quantitative real-time PCR (QRT-PCR)**

Total RNA was extracted from the plasma samples using Y TZOL (Yekata Tajhiz Azma Company, Iran) in accordance with the company's protocol. The quality and quantity of the extracted RNA were determined by electrophoresis on gel agarose and NanoDrop spectrophotometer, respectively. Thereafter, cDNA was synthesized by reverse transcription of the extracted RNA using oligo dT primers and the Hyperscript first-strand synthesis kit (Geneall Biotechnology, Korea). Next, QRT-PCR reaction was performed using the Real Q Plus 2x Master Mix Green Kit (Ampliqon A/S, Denmark) by applying specific primers (*HOTTIP*: Forward sequence 5'AGCTCTTTTCCCCGACA-GTG3', Reverse sequence: 5'CCTTCACCAAGC-TCCCTCTG3', and *GAPDH*: Forward sequence: 5'CATCAAGAAGGTGGTGAAGCAG3', Reverse sequence: 5'GCGTCAAAGGTGGAGGAGTG3') in duplicate. Primers were designed with Gene runner software and their specificity was evaluated by NCBI primer-BLAST. QRT-PCR and data collection was performed by Step One Plus™ real-time PCR system (Thermo Fisher scientific, USA). The results were normalized against *GAPDH* and relative expression of *HOTTIP* was calculated using the *2*^-∆∆CT^ method (15).

**Table 1:** Association between lncRNA *HOTTIP* expression and clinico-pathological data in CRC

**Variables**	**Number of cases**	**% of patients**	**Mean rank**	**P-value**
**Age (year)**				
>50 <50	39 11	78 22	25.49 25.55	0.9**
**Gender**				
Male Female	28 22	56 44	26.89 23.73	0.4**
**Stage**				
I II III	15 18 16	30.6 36.7 32.7	22.73 24.28 27.94	0.5*
**Grade**				
I II III	23 23 4	46 46 8	25.52 24.39 31.75	0.6*
**Tumor location**				
Rectum Sigmoid Rectosigmoid Colon and cecum	30 6 7 7	60 12 14 14	24.57 31.83 29.00 20.57	0.3*
**Histology**				
Adenocarcinoma Mucinous (Colloid) Adenocarcinoma	44 6	88 12	25.91 22.50	0.5**
**Tumor Size**				
>5 cm <5 cm	17 33	34 66	22.24 27.18	0.2**
**Family history**				
Yes No Unknown	17 27 6	34 54 12	32.35 21.07 26	0.04*
**Smoking**				
Non-Smoker Smoker unknown	32 6 10	64 12 20	26.16 20.67 21.50	0.5*
**Drinking**				
Alcoholic Non-alcoholic unknown	3 34 11	6 68 22	22 25.24 22.91	0.8*

* Kruskal- Wallis test; ** Mann-Whitney test

**Statistical analysis**

For describing the quantitative variables mean ± SD was used, and qualitative variables were described by percent. Kolmogorov-Smirnov test was used to evaluate normal distribution of the quantitative measures. Mann-Whitney nonpa-rametric test was used to compare lncRNA *HOTTIP* expression between two groups. Kruskal-wallis and Mann-withney tests were used to investigate the relationship between *HOTTIP *expression and clinicopathological data of the patients. Statistically, P value <0.05 was supposed significant. The sensitivity and specificity of *HOTTIP* dysregulation in a CRC and healthy individual’s plasma was measured by receiver operating characteristic (ROC) analysis. Statistical analysis was performed using SPSS software 23.

## Results

**Overall up regulation of** ***HOTTIP*** **in CRC**

**Fig. 1:**
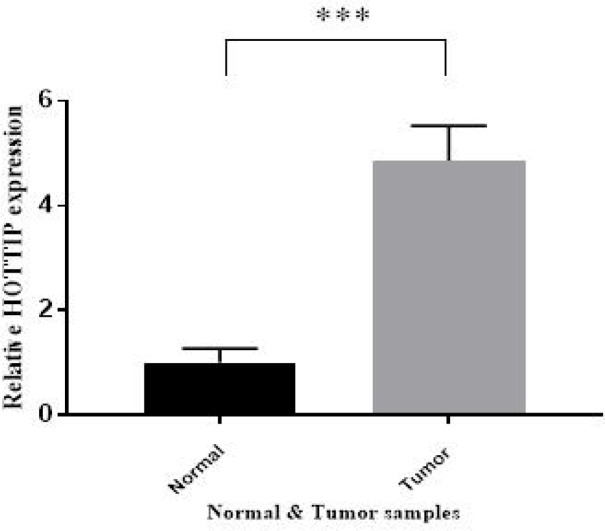
Plasma HOTTIP expression. Overall 4.8 fold higher expression of HOTTIP in CRC plasma samples was observed in comparison with of normal plasma samples (***P< 0.001).

As shown in Figure 1, significant up regulation of *HOTTIP* was observed in plasma samples of CRC patients in comparison with normal group (P <0.001). According to the 2^-∆∆CT ^method results, data greater and less than 1 was considered up and down regulation, respectively. Higher expression of *HOTTIP* was obtained in 39 cases of tumor group (78%), individually.

**Fig. 2:**
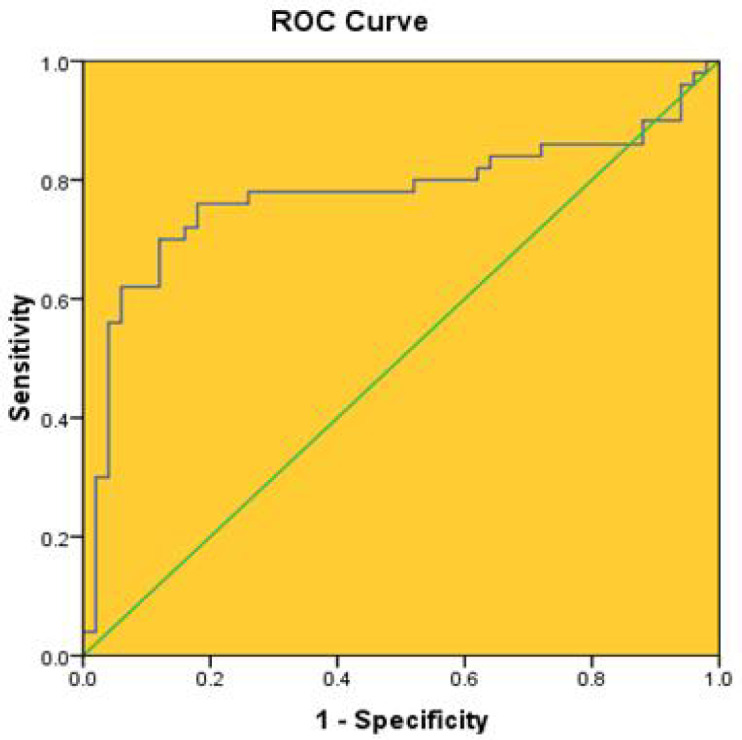
Receiver operating characteristic (ROC) curve for HOTTIP to distinguish CRC disease from healthy persons. The area under the ROC is 0.77

**Fig. 3:**
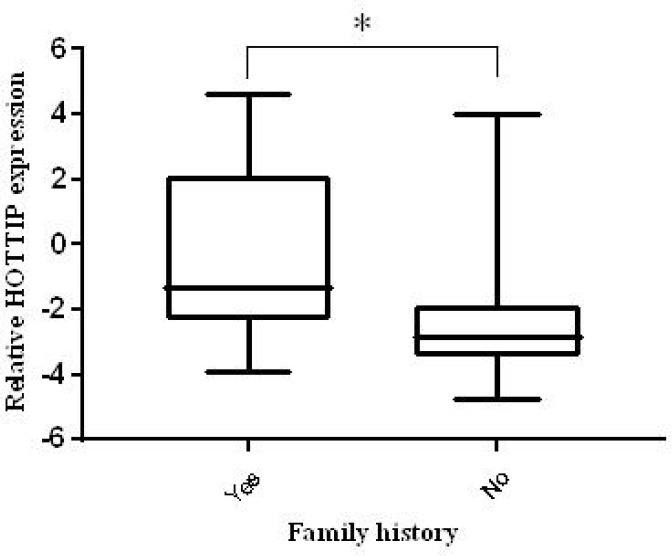
Plasma HOTTIP expression according to family history. HOTTIP expression was significantly higher in patients with family history of CRC (* P < 0.05).

Diagnostic value of plasma *HOTTIP* in CRC patients was assessed by ROC curve analysis. As illustrated in Figure 2, the area under curve (AUC) of the *HOTTIP* in patients with CRC and control people is equal to 0.775 ± 0.05.

**Relationship between** ***HOTTIP*** **expression level and clinico-pathological characteristics of CRC patients**

In this study, there was a significant relationship between the *HOTTIP* expression level and family history of CRC (Figure 3). No significant association was found between *HOTTIP* dysregulation and other clinico- pathological features such as tumor stage, grade, location and size. The other information are presented in Table1.

## Discussion

LncRNAs, a class of RNA molecules with a length of more than 200 nucleotides, are transcribed in the genome and function as regulators in various biological processes. Abnormal expression of this group of molecules participate in various cancers development including CRC (1, 4). LncRNAs may be considered as therapeutic targets and promising biomarker for cancer prognosis (16), and one of well- known lncRNAs with oncogenic role in solid tumors is *HOTTIP* (17).

In present survey we report that *HOTTIP* is significantly over expressed in CRC plasma samples. These data are similar to other studies (13, 18). Unlike similar published researches, the value of our study is the evaluation of target gene in matched biological samples rather than tissue samples. Also, all study subjects have not received any treatment prior to sample collection, had no history of chemoradiotherapy due to cancer with other origins, and no systemic disease. In other words, the confounding factors were excluded while the majority of previous surveys have introduced adjacent non-malignant tissues in their study as a control group and have not clarified exclusion criteria, exactly (13, 18-20). Because microscopic adjacent non- malignant cells may have tumoric cell features at molecular level (21), so this is a drawback to this kind of study design.

Our study did not prove any significant statistical relationship between *HOTTIP* expression and clinical and pathological data, especially tumor stage and size. However, expression level of *HOTTIP* in grade 2 was higher than the first degree.

Contrary to our results, a positive correlation of higher *HOTTIP* with T stage and larger tumor size have been reported by Ren et al. study which was conducted on 156 CRC tissues and 21 adjacent non-malignant tissues (13) as well as another investigation which was carried out on 48 paired CRC and adjacent non-tumor colorectal samples (20). However, the later study indicate no relevance of *HOTTIP* dysregulation to the site and grade of the tumor, lymph nodes metastasis, gender and age (20).

Although, a systematic review and meta-analysis has introduced *HOTTIP* as a lymph node metastasis indicator in human cancers including CRC, but the authors state that their results may be influenced by ethnic bias because the majority of studies have been conducted on population samples in China (22). Therefore, it is expected to observe the racial differences between Iranian ethnicity and other ethnicities. These inconsistent consequences can be related to low sample size of present and previous studies, different design procedure, and different inclusion and exclusion criteria consideration, too.

In order to discover screening biomarkers for CRC, Zhao et al. evaluated the clinical importance of two HOX transcript antisense RNA (*HOTAIR*) and colon cancer associated transcript 1 (*CCAT1*) lncRNAs in plasma samples. They observed increased expression of these two molecules in the plasma of patients. Also, they described that *CCAT1* alone, as a diagnostic biomarker for CRC, has 75% sensitivity and 85% specificity (23). Our data illustrated 82% specificity, and 76% sensitivity for plasma *HOTTIP*. Thus, it is highly suggested to evaluate these two transcripts, *CCAT1* and *HOTTIP*, in a single study because these two transcripts seems to have key properties of novel potential diagnostic biomarkers in combination with each other or one of these transcript alone.

Molecular mechanism of action of *HOTTIP* in CRC development has been little known, and studies on this lncRNA are in the primary stages. In recent years, some studies have tried to elucidate the real role of *HOTTIP* in tumor behaviors including: a survey that suppressed *HOTTIP* expression by siRNA and elucidated that tumor cell migration and invasion *in vitro* are inhibited. This study implies that *HOTTIP* is somehow involved in tumor metastasis through down regulation of dickkopf WNT signaling pathway inhibitor 1 (*DKK1*) tumor suppressor gene (18). Also, apoptosis was induced and cell proliferation was suppressed by knockdown of *HOTTIP* in HCT-116 and SW620 cell lines through targeting serum- and glucocorticoid-inducible kinase 1 (*SGK1*) gene. Besides, it inhibits *GSK3β*, β-catenin, cellular myelocytomatosis oncogene (*C-MYC*), vimentin and matrix metalloproteinase 7 (*MMP-7*) expression, and results in up-regulation of E-cadherin (19). In addition, *HOTTIP* participates in CRC cell growth via silencing p21, a cyclin-dependent kinase inhibitor (20, 24).

As mentioned earlier, there is still no comprehensive information available about *HOTTIP* expression which confirms its application as a biological marker in the diagnosis and treatment of CRC. Thus, functional consequences of altered expression of this lncRNA can be resulted from more extensive examinations.

Our results suggest that higher expression level of *HOTTIP* has the potential to be a diagnostic marker for CRC patients.
